# Transcription factors β-catenin and Hex in postnatal development of the rat adrenal cortex: implication in proliferation control

**DOI:** 10.1016/j.heliyon.2021.e05932

**Published:** 2021-01-09

**Authors:** Natalya V. Yaglova, Dibakhan A. Tsomartova, Sergey S. Obernikhin, Svetlana V. Nazimova, Marina Y. Ivanova, Elizaveta V. Chereshneva, Valentin V. Yaglov, Tatiana A. Lomanovskaya

**Affiliations:** a Laboratory of Endocrine System Development, Federal State Budgetary Institution Research Institute of Human Morphology, Moscow, Russia; b Department of Histology, Cytology, and Embryology, Federal State Funded Educational Institution of Higher Education I.M. Sechenov First Moscow State Medical University, Moscow, Russia

**Keywords:** Adrenal cortex, Postnatal development, Transcription factors, Hex, β-catenin, Wnt signaling, Proliferation, Hormones, Morphology

## Abstract

Transcriptional regulation of growth, maturation, and cell turnover in adrenal cortex during postnatal development has been significantly less studied than in embryonic period, while elucidation of factors mediating its normal postnatal morphogenesis could clarify mechanisms of tumorigenesis in adrenal cortex. Expression of transcription factors Hex, β-catenin, and Wnt signaling in the adrenal cortex of male pubertal and postpubertal Wistar rats were examined. Adrenal cortex morphology and hormone production during postnatal development were also studied. Adrenocortical zones demonstrated similar reduction of Ki-67-expressing cells, but different patterns of morphological and functional changes. Age-dependent decrease in percentage of cells with membrane localization of β-catenin and stable rate of cells with nuclear β-catenin, indicative of Wnt signaling activation, were revealed in each cortical zone. Nuclear β-catenin was not observed in immature areas of zona fasciculata. No association between Wnt signaling activation and rates of proliferation as well as changes in secretion of adrenocortical hormones was observed in postnatal development of rat adrenal cortex. Hex, known as antiproliferative factor, showed up-regulation of expression after puberty. Strong inverse correlations between ratio of Hex-positive cells and proliferating cells were found in zona glomerulosa and zona fasciculata. Zona reticularis demonstrated moderate correlation. Thus, these findings suggest a role for Hex in proliferation control during postnatal development of the rat adrenal cortex and possible implication of Hex down-regulation in adrenocortical dysplasia and neoplasia, which requires further study. Evaluation of Hex expression may also be considered a potent tool in assessment of cell proliferation in rat adrenal cortex.

## 1 Introduction

Transcriptional regulation of postnatal growth, maturation, and cell turnover still remains unclear. Recent data suggest that embryonic transcription factors can have various effects on tissue and organ development before and after birth [1]. Lack of knowledge on embryonic transcription factors action in adult organism complicates investigations of postnatal development and disease. Aberrant expression of some transcription factors regulating organ development is known to promote hyperplasia and neoplasia [2,3], that is why investigation of tumorigenesis requires elucidation of transcriptional factors mediating normal postnatal development. The adrenal cortex produces three classes of steroid hormones which are essential for maintenance of mineral and glucose homeostasis, immune response and sexual maturation [4]. Nowadays, mechanisms regulating postnatal development of the adrenal cortex and factors implicated in adrenocortical cell renewal are just beginning to be identified.

Establishment of functional zonation and regulation of growth is actively studied for last decades, but most research focuses on fetal and perinatal periods [5,6,7]. The Wnt pathway is an evolutionarily conserved signaling pathway involved in cell self-renewal, morphogenesis and development [8]. Recent studies have shown a key role of the Wnt/b-catenin pathway in formation of zona glomerulosa (ZG) and differentiation of aldosterone-producing cells in the adrenal cortex of mice [9,10] and humans [11] by stimulation of angiotensin II receptor and aldosterone synthase expression [12]. The role of canonical Wnt/β-catenin signaling pathway in development and physiology of zona fasciculata (ZF) is poorly understood since the data are sparse and inconsistent. Some investigations observed lack of detectable levels of β-catenin in ZF of adult mice [9]. Further research has showed that non-proliferating fasciculata cells express β-catenin, and Wnt signaling activation inhibits steroidogenesis in mouse ZF [13]. These data are consistent with the results of other investigations found that activation of β-catenin in presumptive fasciculata cells induces ectopic expression of enzymes associated with increased aldosterone production [9,14]. Altogether, these observations show that canonical Wnt/b-catenin signaling pathway promotes formation and functional maturation of ZG and prevents differentiation of ZF in mice. Some recent reports provide evidence for alternative concept, since they demonstrate that β-catenin/Wnt signaling inhibition decreases glucocorticoid levels in mouse adrenocortical cancer cells [15]. The most poorly studied aspect of Wnt signaling pathway in adrenal cortex is temporal expression and activation of β-catenin in zona reticularis (ZR), the innermost layer of the adrenal cortex. Moreover, it should be noted that the most investigations were performed on mice adrenals or mice adrenocortical cell lines while transcriptional regulation of rat adrenal morphogenesis and function are significantly less studied. It is noteworthy, that most experiments on modulation of Wnt signaling in the adrenal cortex were performed on genetically engineered animals and on gain-of-function and loss-of-function models [16,17,18]. It significantly complicates evaluation of Wnt signaling function in wild-type laboratory animals, since transcriptional regulation represents a network of cooperating factors, and impaired function of one regulator may change activity of other factors.

Since activation of canonical Wnt/β-catenin signaling is associated with acceleration of proliferation and even tumorigenesis in the adrenal cortex [15,19,20,21], investigation of Wnt signaling activation in postnatal development requires concomitant assessment of antiproliferative factors. Transcription factors involved in suppression of adrenal cell proliferation and termination of growth are less studied. A homeobox gene named Haematopoietically expressed homeobox (Hex) was identified in haemopoietic cells in 1990th [22]. Investigations have showed that Hex acted with β-catenin in regulation of anteroposterior patterning [23]. Hex expression is required for development of liver, heart, thyroid, thymus, and forebrain during embryogenesis [24,25,26]. Hex has been found to express in different types of cells in the adult [27,28]. The profile of genes repressed or activated by Hex indicates its involvement in regulation of proliferation in adult organism [29,30]. Activation of transforming growth factor-β co-receptor gene expression and repression of genes encoding endothelial cell-specific molecule 1 (ESM1), vascular endothelial growth factor A (VEGFA) and its receptors, demonstrate antiproliferative and antimetastatic potential of Hex [31]. Lowered Hex expression in poorly differentiated tumor cells compared to well-differentiated ones proves association of Hex expression with low level of cell proliferation [32]. Role of Hex in regulation of adrenal cortex development during prenatal and postnatal ontogeny is obscure. We supposed that Hex might be involved in proliferation control during postnatal development of the adrenal cortex. Rat adrenal cortex intensively grows in pubertal period and reaches maximal development at the age of 70 days [33]. We aimed our investigation at elucidation of Hex and β-catenin postnatal expression, Wnt signaling pathway activation in the three adrenocortical zones and association of these factors with morphological and functional changes during critical periods of adrenocortical development.

## 2 Materials and methods

### 2.1 Animals

Male Wistar rats (n = 40) were obtained from Scientific Center of Biomedical Technologies of Federal Medical and Biological Agency of Russia. The animals were housed at +22–23 °C with a 12/12-hr light-dark cycle and given a pelleted standard chow ad libitum. The rats were sacrificed by zoletil overdosage at 42^nd^ and 70^th^ day of age at 9–10.00 AM. 42^nd^ day of postnatal development (PND42) is pubertal age after adrenarche and prior to gonadarche, when adrenals actively grow, and the 70^th^ day postnatal development (PND70) is a time of maximal development of adrenal cortex and termination of growth [33]. The blood and the adrenal glands were collected. Animal procedures were approved by Ethics committee of Research Institute of human morphology (protocol N8a). The investigation was performed in accordance with the handling standards and rules of laboratory animals as consistent with “International Guidelines for Biomedical Researches with Animals” (1985), laboratory routine standards in the Russian Federation (Order of Ministry of Healthcare of the Russian Federation dated 19.06.2003 No.267) and “Animal Cruelty Protection Act” dated 1.12.1999, regulations of experimental animal operation approved by Order of Ministry of Healthcare of USSR No.577 dated 12.08.1977.

### 2.2 Adrenal histology

The adrenal glands were fixed in Bouen solution. After standard histological processing the tissue samples were embedded in paraffin. Equatorial sections of the adrenals were stained with hematoxylin and eosin. Histological examination included light microscopy and computer morphometry of ZG, ZF, and ZR, performed with “Leica DM2500” light microscope and “ImageScope” software (Leica Microsystems Gmbh, Germany). Surface area, size of adrenocortical cells, number of the cells in 1μm^2^, and diameter of capillaries were measured.

### 2.3 Immunohistochemistry

Immunohistochemical evaluation of β-catenin, Hex, and Ki-67 was performed on paraffin-embedded tissues. After antigen retrieval by boiling with 10mM sodium citrate (pH 6.0) endogenous peroxidase was blocked with Hydrogen Peroxide Block and then endogenous immunoglobulins was blocked Protein Block (Thermo Fisher Scientific, USA) according to manufacturer's protocol. The slides were incubated with primary antibodies to β-catenin (1 : 100, Cell Marque, USA), Hex (1 : 200, Santa Cruz Biotechnology, USA), and Ki-67 (1 : 100, Cell Marque, CA, USA) overnight at 8 °C. The reaction was visualized with anti-mouse/antirabbit “UltraVision LP Detection System” reagent kit (Thermo Fisher Scientific, USA) according to manufacturer's recommendations. The sections were counterstained with Mayer's hematoxylin. After dehydration the sections were mounted in polymer media. Sections processed without primary antibodies were used as negative control for secondary antibodies. No nonspecific reactions were registered.

### 2.4 Quantification of the immunohistochemical reactions

Immunohistochemical reactions were evaluated in ZG, ZF, and ZR. Hex expression was assessed as a percent of cells with immunopositive (brown and dark brown) nuclei. The number of cells with the membrane, cytoplasmic, and nuclear localization of β-catenin was calculated and expressed as the percent of the total number of cell in each zone of the adrenal cortex. Cells with immunopositive membranes and negative cytoplasm and nucleus were considered as cells with membrane localization of β-catenin. Cells with immunopositive cytoplasm and immunonegative nuclei were referred to cells with cytoplasmic localization. Cells with immunopositive nuclei with presence/absence of β-catenin in membranes and cytoplasm were considered as cells with nuclear localization. Translocation of β-catenin into nucleus was considered as a marker of activation of canonical Wnt signaling [34,35]. Rate of cell proliferation was determined by calculation of percentage of Ki-67-positive cells in 1mm^2^ of each zone.

### 2.5 Enzyme-linked immunosorbent assay

Collected blood samples were incubated at room temperature for 30 min. Clotted blood was centrifuged at 2700 rounds per minute for 15 min. Serum was transferred into polypropylene tubes. Mineralocorticoid hormone aldosterone, glucocorticoid hormone corticosterone, and adrenal sex steroids androstendione and estrone were measured for assessment of ZG, ZF, and ZR function, subsequently. Hormone concentrations in serum were determined by enzyme-linked immunosorbent assay according to manufacturer's protocols (Cusabio, China) with “Anthos 2010” microplate reader at 450 nm.

### 2.6 Statistical analysis

The statistical analyses were carried out using the software package Statistica 7.0 (StatSoft, USA). The central tendency and dispersion of quantitative traits with approximately normal distribution were presented as the mean and standard error of the mean (М±m). Quantitative comparisons of independent groups were performed using Student's t test, taking into account the values of Levene's test for the equality of variances, and χ^2^. Associations of the total number of Ki-67- and Hex-positive and Ki-67- and nuclear β-catenin-positive cells in each term of the investigation were analyzed using Pearson correlations test. Differences were considered statistically significant at p < 0.05.

## 3 Results

### 3.1 Developmental changes in the adrenal cortex morphology

**Figure 1:**
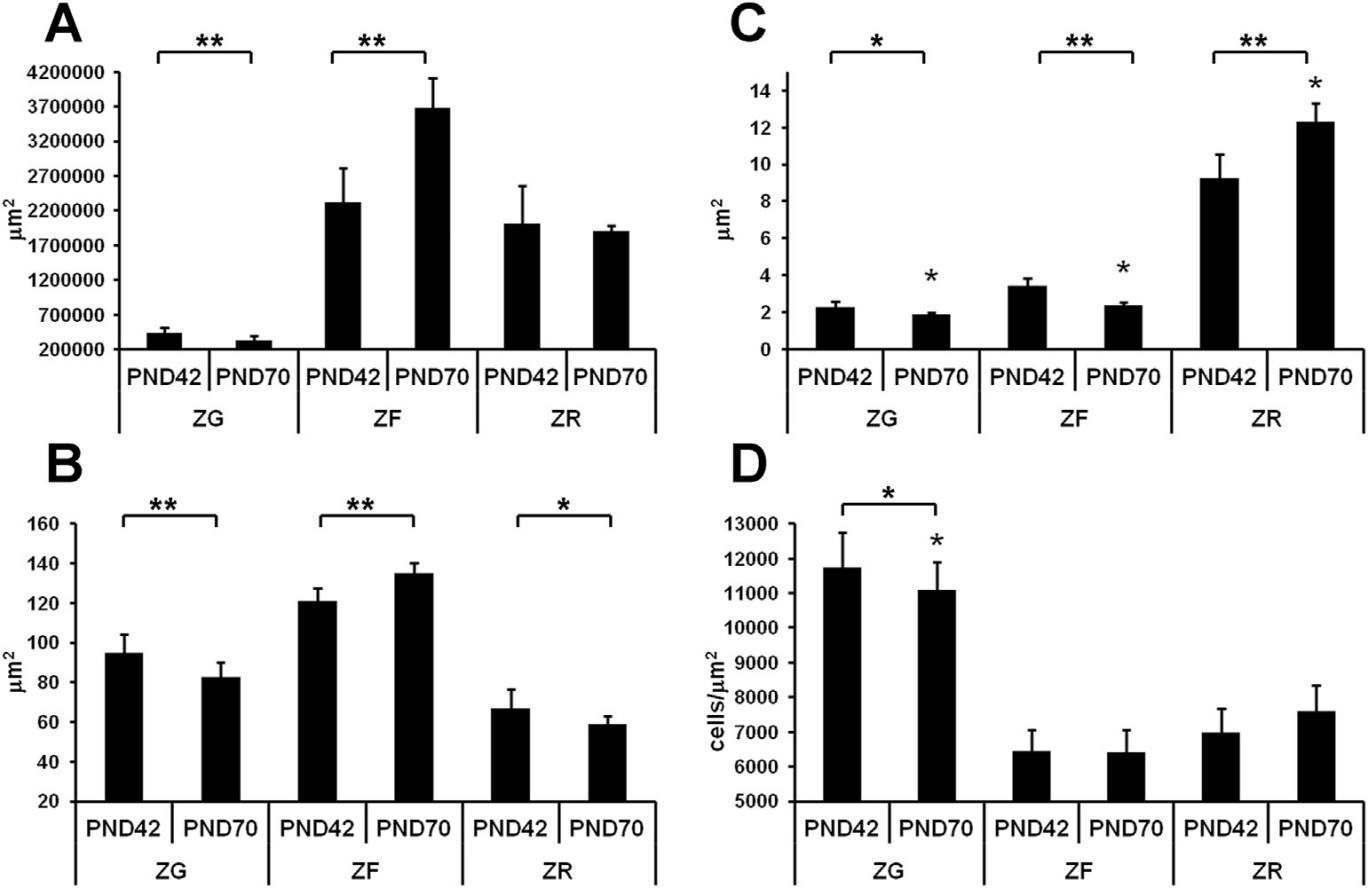
Developmental changes in morphology of rat adrenal cortex. (A) surface area, (B) size of adrenocortical cells, (C) width of capillaries, (D) number of cells in 1μm^2^ of zona glomerulosa (ZG), zona fasciculata (ZF), and zona reticularis (ZR). Data are shown as mean ± S.E.M. PND, day of postnatal development; ∗ p < 0.05; ∗∗ p < 0.01.

The 42^nd^-days old rats exhibited well developed adrenal cortex with three distinguishable concentric layers: ZG, the outermost layer, ZF, and ZR, the innermost layer. Some areas in outer ZF, representing immature developing sites, had not typical bundle structure. Interrupted zona intermedia was observed between ZG and ZF. Intermedia cells demonstrated smaller size and pleomorphic nuclei. Histological examination on the 70^th^ day of postnatal development revealed some changes in structure of the cortex. Diminished area of ZG and enlarged ZF were observed (Figure 1a). Depletion of ZG was associated with reduction of ZG cell size (Figure 1b), narrowing of capillaries (Figure 1c), and decrease in number of cells in 1μm^2^ (Figure 1d). Histological examination of ZF revealed enlarged cells and narrowing of capillaries (Figure 1b, c). No significant differences in cellular density compared to 42^nd^ day were found (Figure 1d). Unlike ZG and ZF, ZR showed no significant changes in surface area (Figure 1a). Only reduction of cell sizes and dilatation of sinusoid capillaries were observed (Figure 1b, c). Cellular density of ZR did not change (Figure 1d).

### 3.2 Developmental changes in adrenocortical cell proliferation

**Figure 2:**
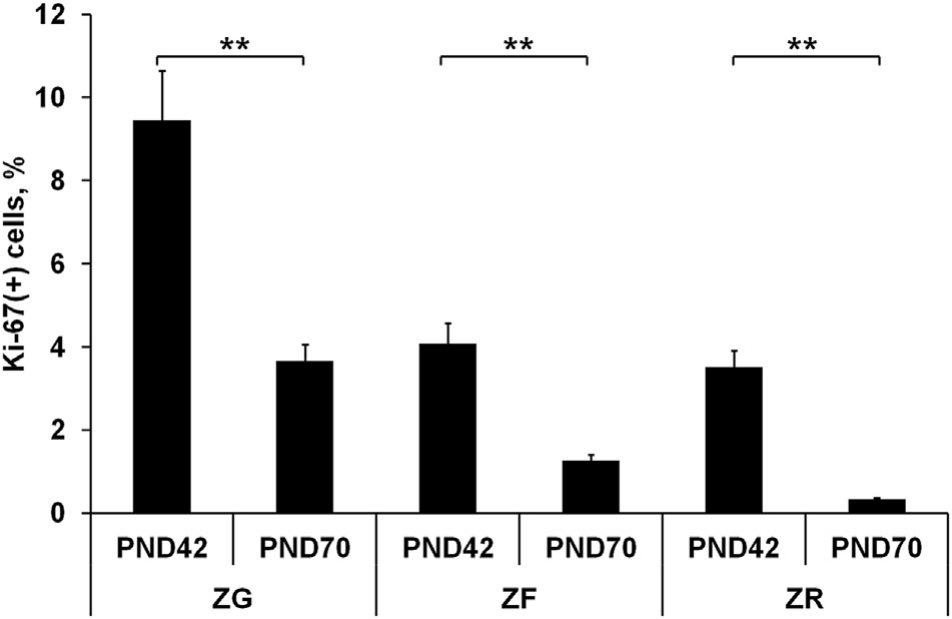
Developmental changes in proliferative activity of adrenocortical cells. Data are shown as mean ± S.E.M. ZG, zona glomerulosa; ZF, zona fasciculata; ZR, zona reticularis; PND, day of postnatal development; ∗∗ p < 0.01.

Ki-67-positive cells were observed throughout the adrenal cortex on the 42^nd^ day of postnatal development. The highest rate of proliferation was observed in ZG. Mitotic activity of ZF cells was 2–3 times lower. The minimal percentage of proliferating cells was found in ZR. This pattern of zone-dependent rate of proliferation remained unchanged with age. On the 70^th^ day cell proliferative activity was significantly lower compared to the 42^nd^ day (Figure 2).

### 3.3 Developmental changes in β-catenin expression in adrenocortical cells

**Figure 3:**
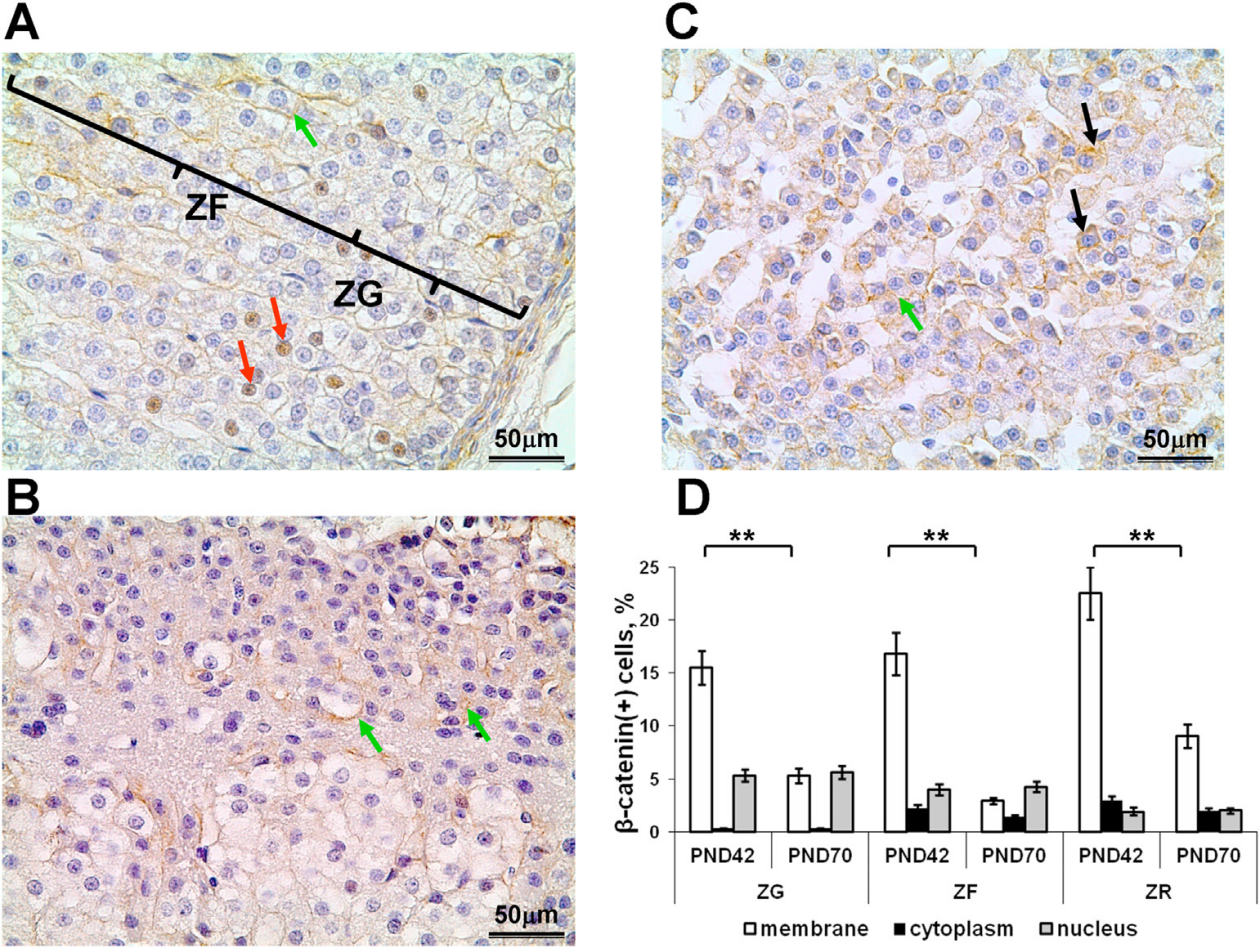
Expression of β-catenin in the adrenal cortex. β-catenin-positive cells on the 42^nd^ day of postnatal development (A) in zona glomerulosa and zona fasciculata; (B) in immature locus of zona fasciculata; (C) in zona reticularis; Immunohistochemical reaction, counterstained with Mayer's hematoxylin. Green arrows indicate membrane, black arrows – cytoplasmic, red arrows – nuclear localization of β-catenin. Scale bar = 50μm. Mag.400. (D) developmental changes in percentage of cells with membrane, cytoplasmic, and nuclear subcellular localization of β-catenin. Data are shown as mean ± S.E.M. ZG, zona glomerulosa; ZF, zona fasciculata; ZR, zona reticularis; PND, day of postnatal development; ∗∗ p < 0.01.

On the 42^nd^ day of postnatal development β-catenin-positive cells were observed in all the zones of the adrenal cortex (Figure 3a,c). The percentages of β-catenin-expressing cells in ZG, ZF, and ZR were similar to each other. Examination of immunopositive cells found prevalence of membrane localization of β-catenin. Cytoplasmic localization was rarely observed. Translocation of β-catenin to nucleus demonstrated its maximal rate in ZG and minimal one in ZR (Figure 3d). Single positive cells, mainly with membrane localization of β-catenin, were observed in the immature areas of ZF (Figure 3b). Zona intermedia demonstrated extremely low number β-catenin-expressing cells and only with cytoplasmatic localization of β-catenin.

Down-regulation of β-catenin expression in all functional zones of the adrenal cortex was found on the 70^th^ day of postnatal development. It was associated with reduction of β-catenin content in cell membranes. Changes in percentage of cells with cytoplasmic localization of β-catenin after termination of adrenal cortex growth were not significant. Percentage of cells with nuclear β-catenin, indicative of Wnt signaling activation, did not change with age (Figure 3d).

### 3.4 Developmental changes in Hex expression in adrenocortical cells

**Figure 4:**
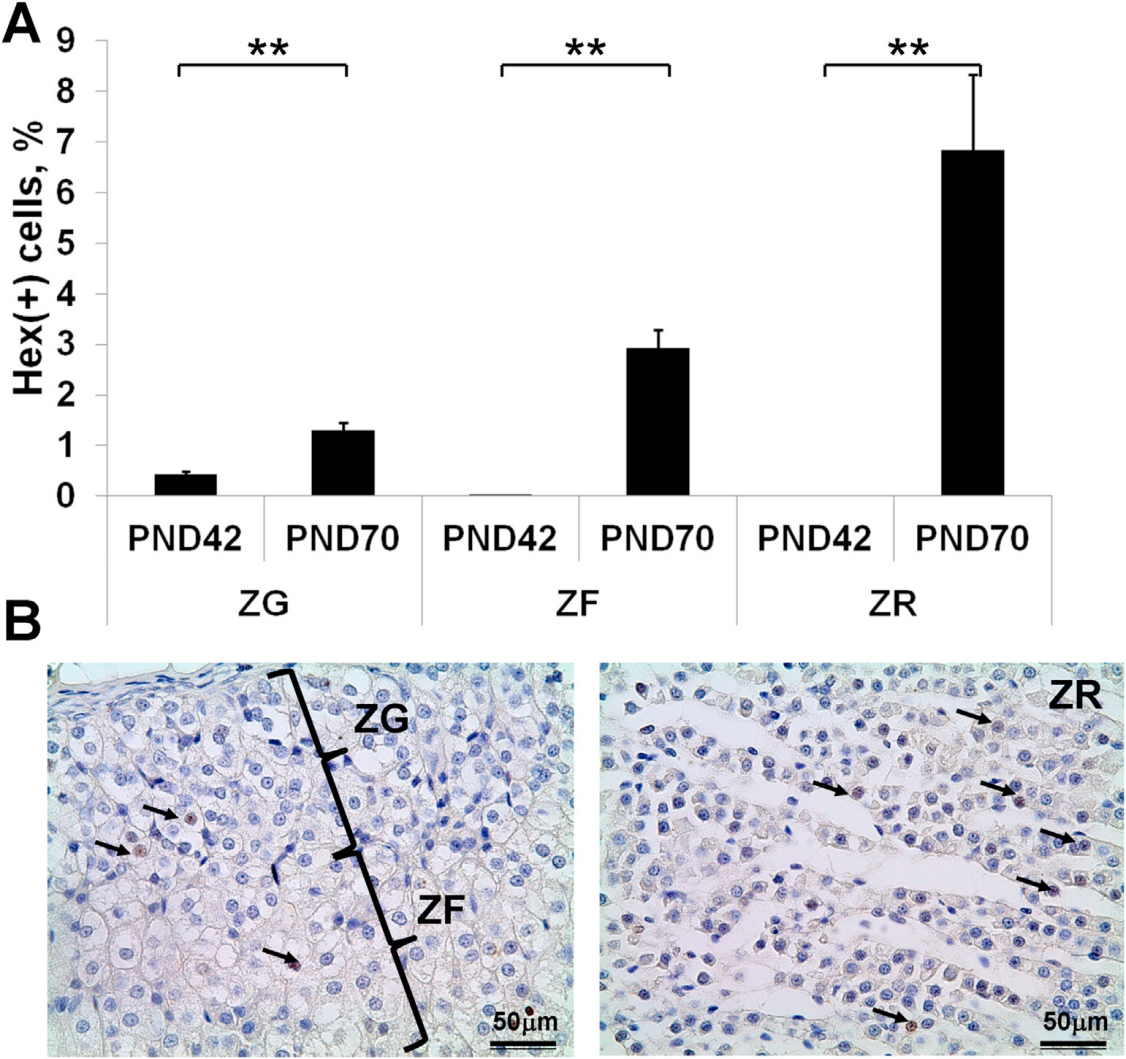
Expression of transcription factor Hex in adrenal cortex. (A) Developmental changes in percentage of Hex-positive adrenocortical cells. Data are shown as mean ± S.E.M. (B) Expression of Hex in adrenocortical zones on the 70^th^ day of postnatal development. Immunohistochemical reaction, counterstained with Mayer's hematoxylin. Hex-positive cells are indicated with arrows. Scale bar = 50μm. Mag.400. ZG, zona glomerulosa; ZF, zona fasciculata; ZR, zona reticularis; PND, day of postnatal development; ∗∗ p < 0.01.

Number of Hex-expressing cells in adrenal cortex was low at the age of 42 days. Up-regulation of Hex expression was revealed on the 70^th^ day of postnatal development (Figure 4). Typical nuclear localization of Hex was observed (Figure 4b). Unlike nuclear β-catenin- and Ki-67- positive cells maximal percentage of Hex-expressing cells was found in ZR, minimal – in ZG. Analysis of association revealed strong inverse correlation between proliferative activity and expression of Hex in ZG and ZF and modern inverse correlation in ZR both in the period of active growth and after its termination. No significant correlation between parameters of proliferation and Wnt signaling activation were found (Table 1).

**Table 1:** Correlation analysis of Ki-67-positive cells and Hex-expressing cells, Ki-67- and nuclear β-catenin-positive cells in adrenocortical zones.

Age	Zones of the adrenal cortex	Hex		Nuclear β-catenin	
		r	p-Value	r	p-Value
42	Zona glomerulosa	-0.91	0.0003	-0.41	0.24
	Zona fasciculata	-0.93	0.00001	0.02	0.95
	Zona reticularis	-0.77	0.009	-0.11	0.75
PND 70	Zona glomerulosa	-0.90	0.0003	0.37	0.29
	Zona fasciculata	-0.80	0.005	-0.30	0.41
	Zona reticularis	-0.81	0.005	0.40	0.25

### 3.5 Developmental changes in functional activity

**Figure 5:**
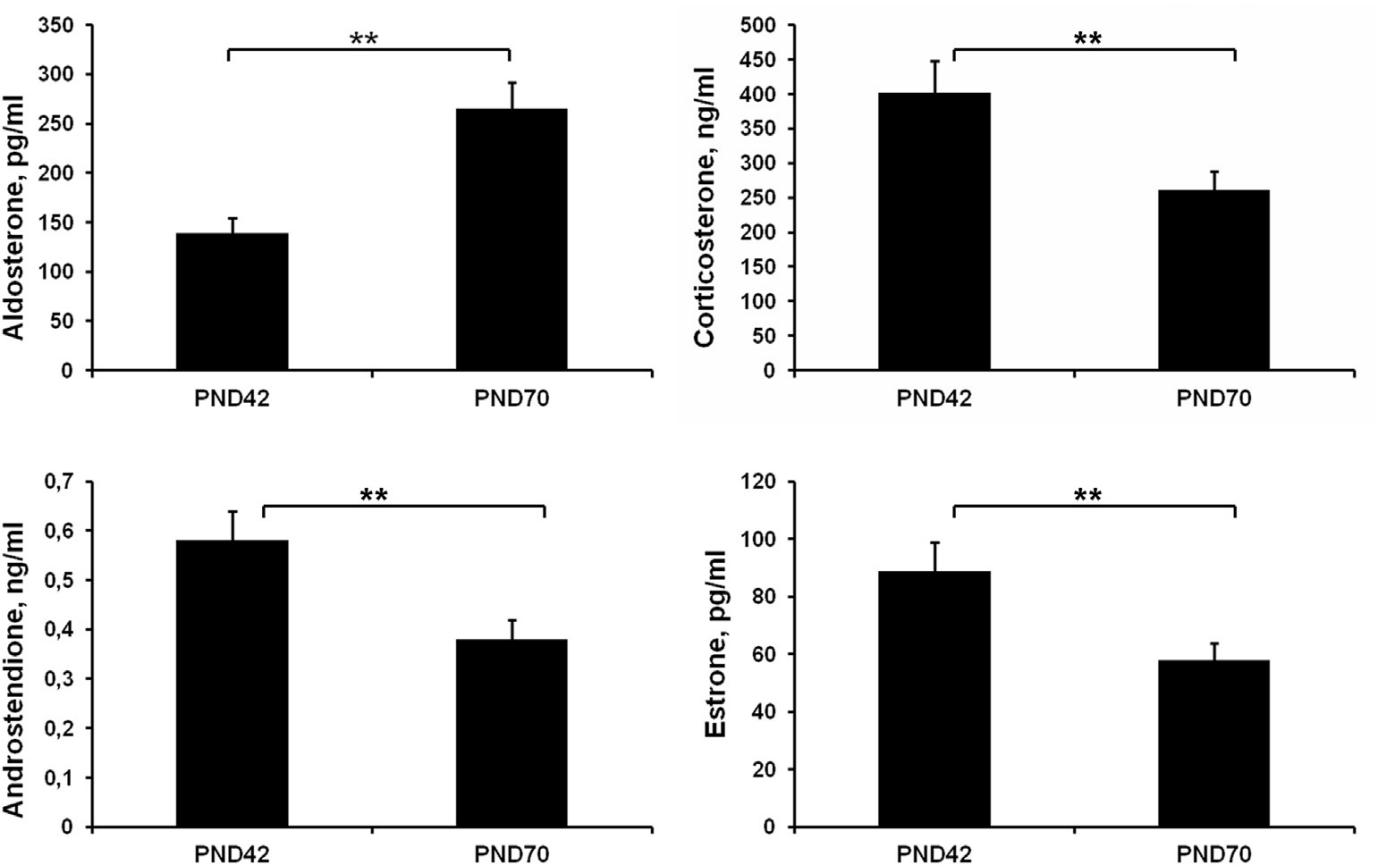
Developmental changes in production of adrenocortical hormones. Data are shown as mean ± S.E.M. PND, day of postnatal development; ∗∗ p < 0.01.

ZG demonstrated increased production of aldosterone after puberty (Figure 5). Serum levels of corticosterone and adrenal sex steroids decreased on the 70^th^ day of postnatal development, indicating lowered functional activity of ZF and ZR (Figure 5). Therefore, hormone production changed independently of parameters of canonical Wnt/β-catenin signaling activation in each zone of the adrenal cortex.

## 4 Discussion

Rat adrenal cortex undergoes significant morphological alterations in ontogeny [6]. The present study revealed three different types of morphodynamics during sexual maturation of organism: reduction of ZG, enlargement of ZF, absence of significant changes in area of ZR. Morphometrical assessment proved that reduction of ZG resulted from decrease in number of glomerulosa cells in adrenal cortex, and not from compaction of cells due to reduction of their size and narrowing of capillaries. Enhanced aldosterone production after puberty by reduced ZG indicated increased functional load on the hormone-producing cells. Our previous study showed that ZG cells increased their functional capacity after puberty by reorganization of mitochondrial apparatus, which provides initial steps of steroidogenesis [36,37]. Accelerated secretory activity and reduced cell content are usually associated with more intensive cell renewal [38,39]. In mice subcapsular and glomerulosa cells represent a source for cell renewal. These cells provide maintenance of cell homeostasis in inner layers of the adrenal cortex by proliferation and transdifferentiation [18,19]. Unlike mice rat adrenal cortex has well distinguishable ZG and ZF separated by some layers of undifferentiated cells called zona intermedia. It prevents direct transition of glomerulosa cells into fasciculata ones and provides another source for cell renewal in adrenal cortex. The highest rate of cell proliferation found in the present study suggests that ZG requires higher cell turnover for tissue homeostasis than ZF, which increases cell content during development, and ZR, which function depletes after puberty.

Immunohistochemical evaluation of β-catenin revealed that patterns of its expression in rats and mice differ. In mice β-catenin expression has been found in outer layers of adrenal cortex, mainly in ZG [34]. In rats all the three functional zones of rat adrenal cortex demonstrated expression of β-catenin with membrane, cytoplasmic, and nuclear subcellular localization. Unlike mice rats have demarcated ZG and ZF and clearly defined ZR. It has been shown that periods of active growth and functional maturation of ZG, ZR, and ZF during postnatal development differ in time [33]. Our findings, demonstrating earlier growth of ZG and ZR and later growth of ZF, also confirm these data. More pronounced histological differentiation of adrenocortical zones and discrete time-periods of their growth in rats suggest different from mice mode of transcriptional regulation of adrenal gland development. Termination of adrenal growth on the 70^th^ day of postnatal development was associated with decreased cell proliferation and lowered content of β-catenin in cell membranes in all functional zones. β-catenin in association with *α*-catenin and E-cadherin is known to form adherens junctions [40]. It stabilizes cell-cell-adhesions, which are essential for normal cell physiology and tissue architecture [41,42,43]. Smaller content of β-catenin in membranes attenuates contact inhibition and, thereby, provides better conditions for proliferation of cells [44,45,46]. Our results demonstrate that age-dependent inhibition of cell proliferation is not associated with higher β-catenin accumulation in cell membranes. Reduction of β-catenin content in cell membranes may have a dual interpretation. Translocation of β-catenin from membrane to cytoplasm may be required for maintenance of Wnt signaling pathway and transcription of target genes [35]. Lower content of membrane-bound β-catenin also results from dissociation of β-catenin-E-cadherin complex. Released β-catenin is known to activate Wnt-independent expression of tissue-specific proteins [47]. So, this dual modality of β-catenin provides gene expression and synthesis of proteins, which regulate cell proliferation and differentiation. Recent data have shown that β-catenin-driven hyperplasia of ZG implicated both proliferation-dependent and proliferation-independent mechanism including block in the ability of ZG cells to transdifferentiate into ZF cells [18]. Probably, reduction of membrane β-catenin content was required for β-catenin interaction with other transcription factors. At the same time, no obvious correlation between changes in percentage of adrenocortical cells with membrane-bound β-catenin and hormone production was observed. This fact lends support to hypothesis that role of β-catenin and Wnt signaling pathway in cell differentiation and regulation of steroidogenesis in ZF and ZR differs from ZG. This idea corresponds with previously found inhibition of steroidogenesis in ZF by Wnt signaling [13]. In early reports Wnt-signaling was usually attributed to ZG [9]. We revealed β-catenin expression throughout adrenal cortex and found its identical changes in functional zones. Remarkably, that higher aldosterone secretion, demonstrated in present study, was not associated with increased number of cells with nuclear localization of β-catenin. Wnt signaling is considered a key factor for early adrenal development and differentiation of ZG, but its role in later growth is obscure [34]. It is likely, that activation of Wnt signaling is not required for enhancement of aldosterone production in highly differentiated cells. Wnt signaling has been found to inhibit development of ZF [9]. Absence of nuclear β-catenin in immature areas of ZF allows to hypothize that activation of Wnt signaling is not essential for cell differentiation and formation of typical tissue structure. Active growth of ZF during puberty without higher rate of translocation of β-catenin to nucleus gives support to this hypothesis. It is noteworthy that cells of zona intermedia, considered as low-differentiated pool for cell renewal [6], did not demonstrate activation of canonical Wnt. ZR is the most poorly studied layer of the adrenal cortex. Its physiological significance diminishes after gonadarche unlike ZG and ZF [33]. Changes in ZR morphology like reduction of cell size and dilatation of blood vessels found in the present study indicate early stage of age-dependent regression. Our study revealed that ZR cells had the highest rate of membrane-bound β-catenin. Lack of cell contacts due to reticular structure and need to maintain tissue integrity are the most trusted causes for increased β-catenin binding to the cell membrane. Age-dependent decline in cell proliferation and hormone secretion in ZR was also associated with constant level of nuclear and decrease in membrane β-catenin rates. Thus, ZR did not show any correlation between parameters of β-catenin expression and Wnt signaling activation and cell proliferation rates and hormone production. These findings suggest that regulation of steroidogenesis in adrenal cortex in adult may differ from embryo and neonate. Species differences are also possible. Identity of age-dependent changes in β-catenin expression among adrenocortical zones also suggests that morphogenetic role of canonical Wnt signaling may differ during ontogeny. Stable rate of nuclear β-catenin indicates that Wnt signaling in adult adrenals may be more required for cell renewal then for growth. This suggestion is supported by shown in the present study absence of cells with β-catenin-positive nuclei in developing ZF and corresponds with other reports showed implication of Wnt signaling in regeneration of adrenal cortex [48,49].

Assessment of Hex expression in developing adrenal cortex revealed correlations between percentage of Hex-positive and Ki-67-positive cells. Higher proliferation rate found in ZG was associated with lowered Hex expression while low proliferating ZR cells demonstrated maximal rate of Hex expression. Significant inverse correlation between Hex-positive and Ki-67-positive cells in each functional zone suggests that initiation of Hex expression results in inhibition of cell proliferation. These data allow to consider transcription factor Hex a potent regulator of adrenal postnatal morphogenesis. Our findings show that Hex exhibits antiproliferative activity in adult rat adrenal cortex as well as in previously reported antiproliferative and antimetastatic action in tumor cell lines [31,32]. Investigations of adrenal carcinogenesis in animals and preclinical trials of novel antitumor preparations require reliable markers and validation tests. Canonical Wnt signaling pathway is known as an inductor of proliferation, and its aberrant signaling is observed in dysplasia and tumors [50]. Disregulation of Wnt signaling also has been shown to initiate adrenocortical tumorigenesis [19,21]. But our findings revealed lower potency of β-catenin expression and Wnt signaling activation as a marker of cell proliferation during postnatal development in contrast to transcription factor Hex, which demonstrated close connection with proliferation rate.

Limitations of the study: The study is limited to normal postnatal development of adrenal cortex in wild-type rats.

## 5 Conclusions

The functional zones of rat adrenal cortex demonstrate different morphological changes, but similar patterns of β-catenin expression and its subcellular distribution during postnatal development. Termination of adrenal growth is associated with reduction in number of cells with β-catenin in outer membrane in the adrenocortical zones. Parameters of β-catenin/Wnt signaling activation do not change after termination of growth, do not correlate with changes in rate of cell proliferation and show no association with functional activity of zona glomerulosa, zona fasciculata, and zona reticularis. Formation of specific histoarchitectonics by fasciculata cells in postnatal development seems not to require activation of Wnt signaling pathway. Unlike β-catenin, expression of transcription factor Hex demonstrates correlation with decreased proliferation activity in all functional zones and may be considered a more potent marker of cell proliferation. Thus, these findings suggest a role for Hex in proliferation control in the rat adrenal cortex during postnatal development and possible implication of Hex down-regulation in adrenocortical dysplasia and neoplasia, which requires further study.

## Declarations

### Author contribution statement

N.V. Yaglova, D.A. Tsomartova, S.S. Obernikhin: Conceived and designed the experiments; Performed the experiments; Analyzed and interpreted the data; Wrote the paper.

S.V. Nazimova, M.Y. Ivanova, E.V. Chereshneva: Performed the experiments; Analyzed and interpreted the data.

V.V. Yaglov: Analyzed and interpreted the data; Contributed reagents, materials, analysis tools or data.

T. Lomanovskaya: Analyzed and interpreted the data.

### Funding statement

This work was supported by the 10.13039/501100012190Ministry of Science and Higher Education of the Russian Federation (No. АААА-А17-117013050048-6).

### Data availability statement

The authors do not have permission to share data.

### Declaartion of interests statement

The authors declare no conflict of interest.

### Additional information

No additional information is available for this paper.
